# Predicting disease-free survival in colorectal cancer by circulating tumor DNA methylation markers

**DOI:** 10.1186/s13148-022-01383-8

**Published:** 2022-12-01

**Authors:** Xin Yang, Xiaofeng Wen, Qin Guo, Yunfeng Zhang, Zhenxing Liang, Qian Wu, Zhihao Li, Weimei Ruan, Zhujia Ye, Hong Wang, Zhiwei Chen, Jian-Bing Fan, Ping Lan, Huashan Liu, Xianrui Wu

**Affiliations:** 1 Department of Colorectal Surgery, The Sixth Affiliated Hospital, Sun Yat-sen University, 26 Yuancun Erheng Rd, Guangzhou, 510655 Guangdong China; 2 Guangdong Provincial Key Laboratory of Colorectal and Pelvic Floor Diseases, The Sixth Affiliated Hospital, Sun Yat-sen University, Guangzhou, 510655 Guangdong China; 3 Department of the General Surgery, Shenzhen People’s Hospital (The Second Clinical Medical College, Jinan University, The First Affiliated Hospital, Southern University of Science and Technology), Shenzhen, 518020 Guangdong China; 4 Department of Pathology, School of Basic Medical Science, Southern Medical University, Guangzhou, 510515 China; 5 AnchorDx Medical Co., Ltd, Unit 502, 3rd Luoxuan Road, International Bio-Island, Guangzhou, 510300 China

**Keywords:** Colorectal cancer, DNA methylation, Disease-free survival, Prognostic prediction

## Abstract

**Background:**

Recurrence represents a well-known poor prognostic factor for colorectal cancer (CRC) patients. This study aimed to establish an effective prognostic prediction model based on noninvasive circulating tumor DNA methylation markers for CRC patients receiving radical surgery.

**Results:**

Two methylation markers (cg11186405 and cg17296166) were identified by Cox regression and receiver operating characteristics, which could classify CRC patients into high recurrence risk and low recurrence risk group. The 3-year disease-free survival was significantly different between CRC patients with low and high recurrence risk [Training set: hazard ratio (HR) 28.776, 95% confidence interval (CI) 3.594–230.400; *P* = 0.002; Validation set: HR 7.796, 95% CI 1.425–42.660, *P* = 0.018]. The nomogram based on the above two methylation markers and TNM stage was established which demonstrated robust prognostic prediction potential, as evidenced by the decision curve analysis result.

**Conclusions:**

A cell-free DNA methylation model consisting of two DNA methylation markers is a promising method for prognostic prediction in CRC patients.

**Supplementary Information:**

The online version contains supplementary material available at 10.1186/s13148-022-01383-8.

## Background

Colorectal cancer (CRC) is the third leading malignancy and the second most common cause of global cancer-related death [1]. Radical surgery is the cornerstone of current therapeutic modalities for localized advanced CRC patients, which has improved their clinical outcomes dramatically. Unfortunately, about a quarter of CRC patients develop recurrence after radical surgery [2, 3]. Worse yet, CRC patients with recurrence have an inferior prognosis, with 60% of these patients suffering from death within 2 years [4].

The combination of imaging examination and serum tumor markers, such as carcinoembryonic antigen (CEA) and carbohydrate antigen19-9 (CA19-9), remains the routine monitoring approach for recurrence detection in CRC patients after radical surgery [5–10]. The sensitivity and specificity for postoperative recurrence and metastasis detection of CEA are 58–89% and 75–98% respectively, while those of CA19-9 range from 26 to 56% and 83 to 87% separately [11, 12]. These data indicated that CEA and CA19-9 have poor performance in prognosis monitoring. Therefore, there is an urgent clinical need to improve the diagnostic efficacy of CRC recurrence after radical surgery, which can remarkably improve the clinical outcomes of these patients.

As an epigenetic mechanism, DNA methylation plays a vital role in tumorigenesis and development [13]. Abnormal methylation in tumors often results in aberrant gene expression [14]. Tumor cells with necrosis and apoptosis can release DNA harboring abnormal methylation; these extracellular nucleic acid fragments are named circulating tumor DNA (ctDNA), which can truly reflect features of cancer [15–17]. Plenty of studies suggest a strong link between ctDNA methylation and tumorigenesis; ctDNA methylation has been utilized as a valuable marker for the early diagnosis of CRC, which was further confirmed by our previous studies [18–28] 29. However, the mechanism of ctDNA methylation in regulating prognosis for CRC patients requires further investigation. This work established an effective prognostic model based on two noninvasive ctDNA methylation markers, which can predict the 3-year disease-free survival (DFS) in CRC patients after radical surgery.

## Results

### Patient demographics and clinical characteristics

**Fig. 1:**
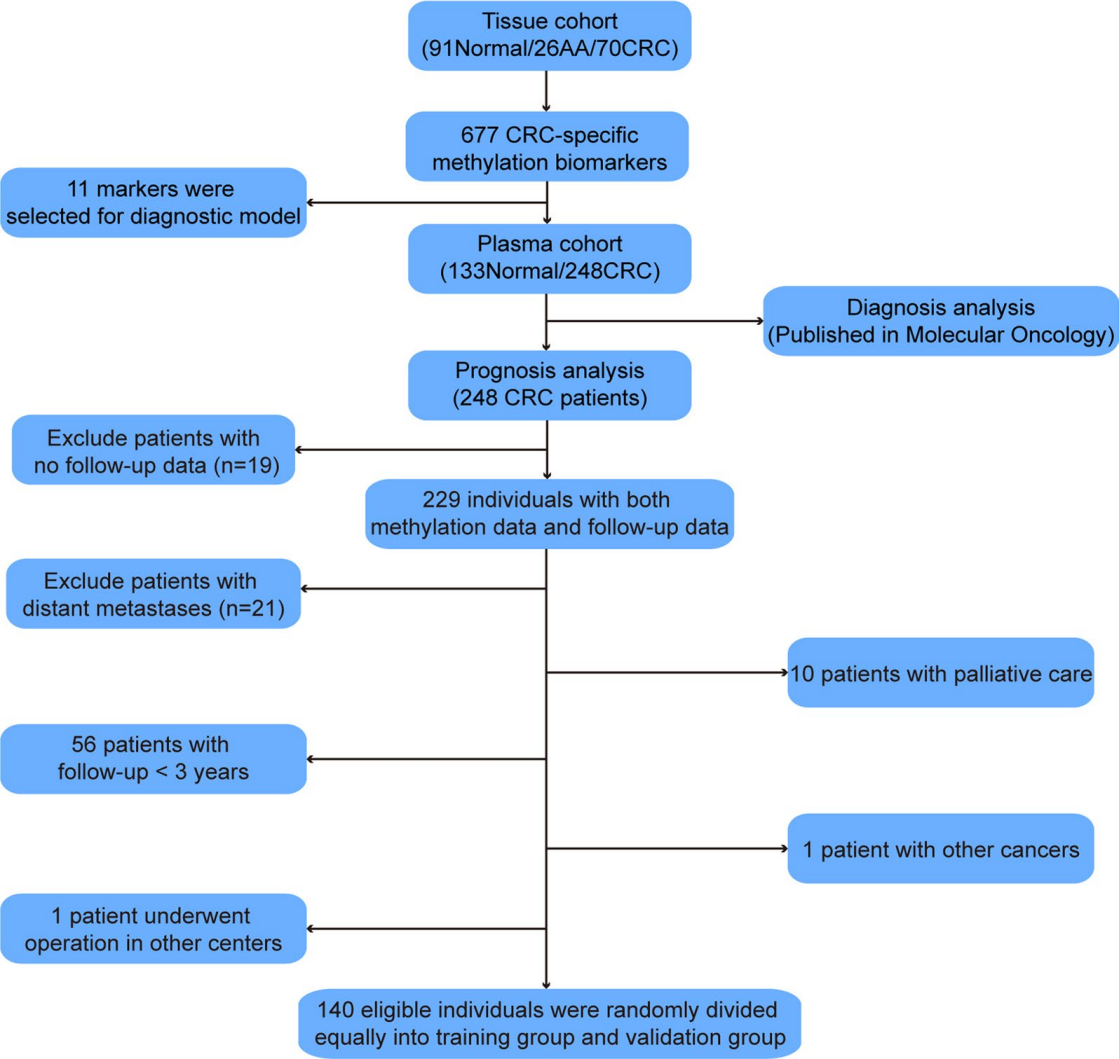
Workflow chart and enrollment of the study

As described in our previous study [29], 248 CRC patients were enrolled between August 2016 and May 2018 to test the contribution of ctDNA methylation for early diagnosis of CRC. In the present study, the 3-year DFS and overall survival (OS) from 84 CRC patients in the training group and 56 patients in the validation group were analyzed (Fig. 1). The clinical characteristics for all patients included are summarized in Table 1. All 140 patients underwent a minimum follow-up of 3 years after radical surgery. Recurrence within 3 years was observed in 9 (10.71%) in the training group and 6 (10.71%) in the validation group. In addition, 5 (5.95%) patients and 3 (5.36%) patients died within 3 years after radical surgery in the training and validation data sets, respectively.

**Table 1:** Clinical characteristics of the entire study cohort

Characteristic	Training group	Validation group	*P*
N	84	56	
Gender, *n* (%)			0.916
Female	35 (25%)	22 (15.7%)	
Male	49 (35%)	34 (24.3%)	
BMI (kg/m^2^), *n* (%)			1.000
18.5–23.9	46 (32.9%)	31 (22.1%)	
≥24, or <18.5	38 (27.1%)	25 (17.9%)	
CEA (ug/L), *n* (%)			0.683
≤ 100	81 (57.9%)	53 (37.9%)	
> 100	3 (2.1%)	3 (2.1%)	
CA199 (U/mL), *n* (%)			0.626
≤37	73 (52.1%)	51 (36.4%)	
>37	11 (7.9%)	5 (3.6%)	
pT, *n* (%)			0.219
T1	14 (10%)	8 (5.7%)	
T2	19 (13.6%)	14 (10%)	
T3	42 (30%)	33 (23.6%)	
T4	9 (6.4%)	1 (0.7%)	
pN, *n* (%)			0.071
N0	66 (47.1%)	37 (26.4%)	
N1	11 (7.9%)	16 (11.4%)	
N2	7 (5%)	3 (2.1%)	
pTNM, *n* (%)			0.229
Stage I	32 (22.9%)	16 (11.4%)	
Stage II	34 (24.3%)	21 (15%)	
Stage III	18 (12.9%)	19 (13.6%)	
Age (years), mean ± SD	59.39 ± 11.22	60.5 ± 9.69	0.535

### Limited contribution of current diagnostic biomarkers for prognostic prediction

**Fig. 2:**
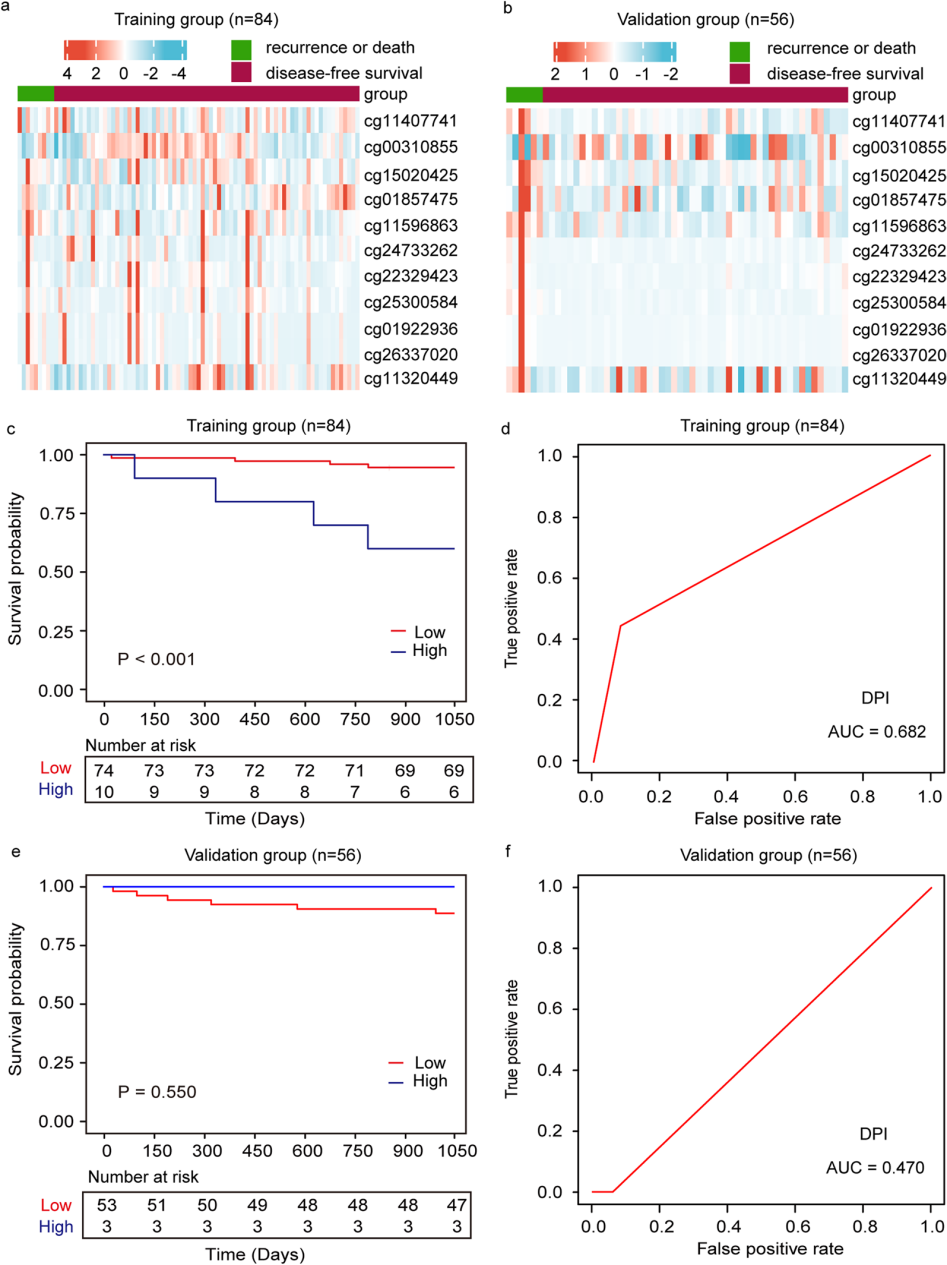
Prognostic prediction of 3-year DFS in CRC patients based on diagnostic markers. Unsupervised hierarchical clustering of the diagnostic markers in the training (**a**) and validation group (**b**). Survival curves of CRC patients with low DPI and high DPI in the training group (**c**) and validation group (**e**). ROC curve and corresponding AUCs for 3-year DFS predicted by DPI in the training group (**d**) and validation group (**f**)

With ctDNA methylation, our previous study has discovered valuable diagnostic markers for the early diagnosis of CRC [29]. Based on this study, we first set out to evaluate the contribution of these diagnostic markers for prognostic prediction. Time-dependent receiver operating characteristic (ROC) [30] analysis indicated that these diagnostic markers exhibited low to medium accuracy in 3-year DFS prediction (Additional file 1: Fig. S1). Moreover, Cox regression analysis was performed on these eleven methylation markers (cg11407741, cg00310855, cg15020425, cg01857475, cg11596863, cg24733262, cg22329423, cg25300584, cg01922936, cg26337020, and cg11320449) in the training group. The coefficients were used to calculate the prognostic index for 3-year DFS based on diagnostic markers (DPI) (Additional file 1: Table S1). Univariate Cox analysis showed that DPI was a significant factor related to 3-year DFS, with a hazard ratio (HR) of 7.413 [95% confidence interval (CI) 1.982–27.730, *P* = 0.003], and the Kaplan–Meier curves indicated that the risk of 3-year DFS was increased with higher DPI in the training group (*P* < 0.001). However, in the validation set, the Kaplan–Meier curves showed that DPI did not correlate with 3-year DFS (*P* = 0.550) (Fig. 2). Together, these results suggested that the diagnostic markers had limited contribution in recurrence prediction, highlighting an unmet clinical need to discover alternative ctDNA methylation markers for prognostic prediction.

### Discovery of ctDNA methylation markers for the 3-year DFS prediction

**Fig. 3:**
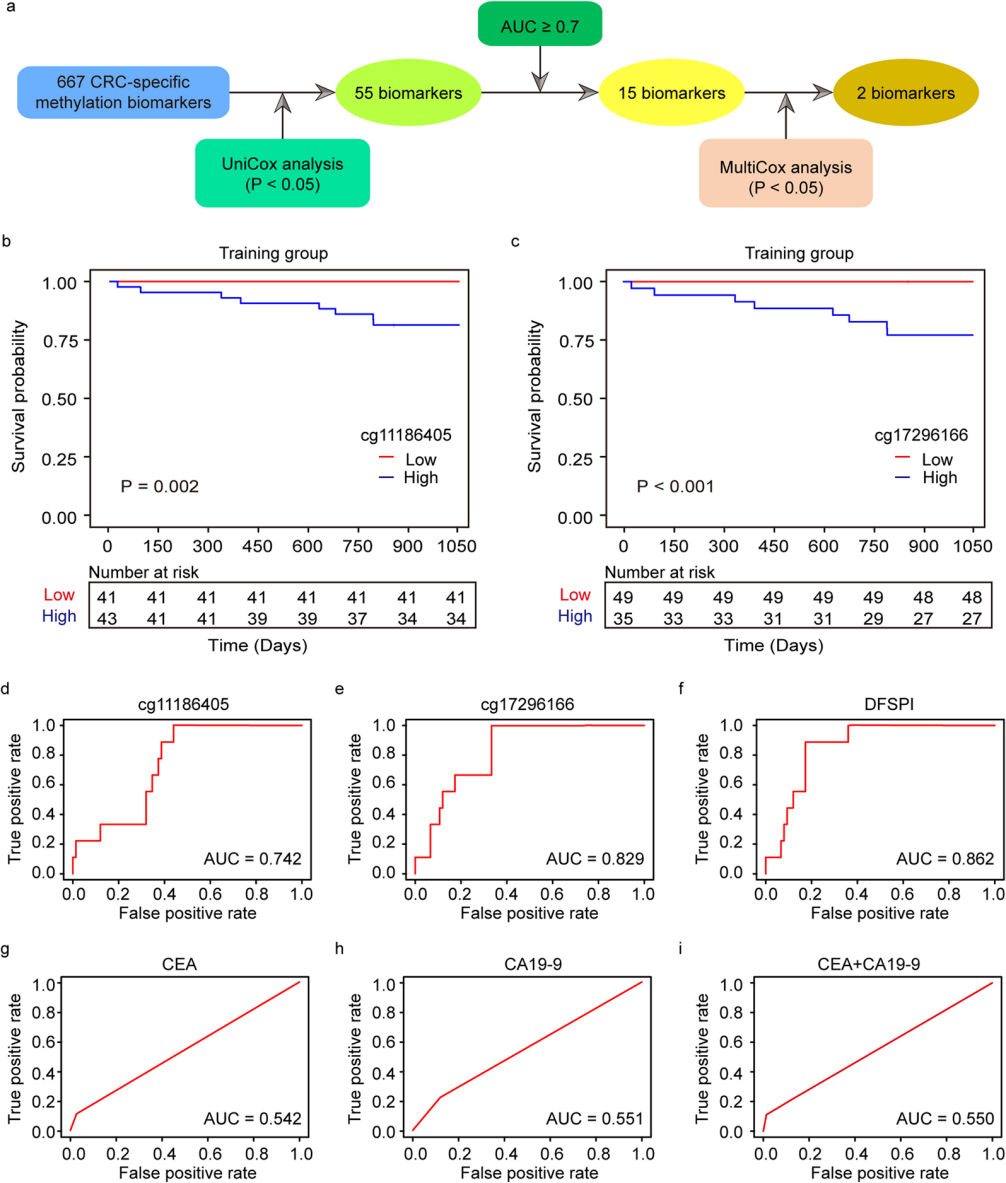
Survival analysis based on selected biomarkers. **a** Workflow for screening methylation markers related to 3-year DFS. **b**–**c** Survival curves of CRC patients with low and high cg11186405/cg17296166 in the training group. **d**–**i** ROC curve and corresponding AUCs for 3-year DFS predicted by cg11186405, cg17296166, DFSPI, CEA and CA19-9

To identify ctDNA methylation markers for 3-year DFS prediction in CRC patients, a modified screening flowchart was used as previously described [31]. Based on a panel of 667 CRC-specific DNA methylation markers in our previous study [29], univariate Cox regression analysis was applied which identified 55 markers with a *P* value < 0.05 (Additional file 1: Table S2). Through time-dependent ROC analysis, 15 out of the 55 methylation biomarkers were identified with their receiver operating characteristic curve (AUC) greater than 0.7. To optimize the predictive model, multivariate Cox regression analysis was employed which showed that cg11186405 and cg17296166 were independent risk factors for 3-year DFS in CRC patients (Fig. 3a). Therefore, these two markers were selected for prognostic predictive model construction. The “Maxstat” package of R was used to generate the optimum cutoff scores for these two markers to classify patients into high-risk and low-risk groups. Kaplan–Meier curves manifested that the increased expression of either cg11186405 or cg17296166 was associated with poorer prognosis (Additional file 1: Table S3 and Fig. 3b, c).

**Fig. 4:**
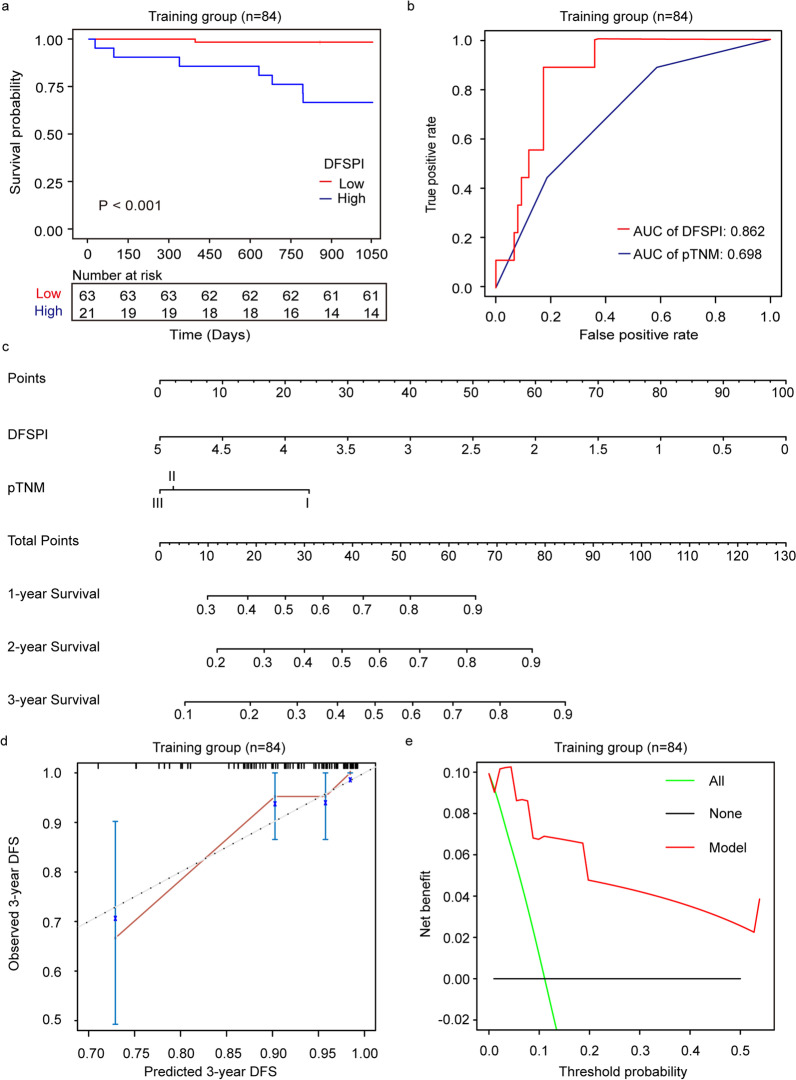
Establishment of prognostic prediction model based on ctDNA methylation markers. **a** Survival curves of CRC patients with low DFSPI or high DFSPI in the training group. **b** ROC and corresponding AUCs for 3-year DFS predicted by DFSPI and pTNM in the training group. **c** Nomogram for predicting DFS of CRC patients based on DFSPI and pTNM. **d** Calibration plot of nomogram for predicting three-year DFS in the training group. **e** Decision curve analysis of the prognostic prediction model

A new prognostic index based on 3-year DFS (DFSPI) was built using the above two markers. And time-dependent ROC analysis showed that DFSPI was better than the individual ones (Fig. 3d–f). As the routine monitoring biomarker in CRC patients, CEA, CA19-9, and a combination of these two markers both were demonstrated with poor performance in postoperative recurrence detection (Fig. 3g–i). Univariate Cox regression analysis revealed that DFSPI was significantly associated with 3-year DFS in CRC patients, with HR as 28.776 [95% CI 3.594–230.400, *P* = 0.002] (Table 2). Based on this cutoff, the Kaplan–Meier curve indicated that the median survival time in CRC patients with low risk was significantly longer than those with high-risk (*P* < 0.001) (Fig. 4a).

**Table 2:** Univariate Cox regression analysis with covariates including DFSPI, and clinical characteristics for 3-year disease-free survival in the training group

Factor	Coef	Exp(coef)	Se(coef)	*z*	*P*
DFSPI	3.36	28.78	1.06	3.17	**0.002**
Age	0.04	1.04	0.03	1.78	0.240
Gender	− 1.06	0.35	0.71	− 1.50	0.133
BMI	− 1.11	0.33	0.80	− 1.38	0.168
CEA	1.21	3.34	1.06	1.34	0.256
CA19-9	0.68	1.97	0.80	0.85	0.397
pT	1.48	4.40	0.53	2.78	**0.006**
pN	0.72	2.05	0.40	1.82	0.069
pTNM	0.94	2.57	0.46	2.03	**0.043**

The bold fonts indicate the *P* value ≤ 0.05

*Coef* The regression coefficients; *Exp(coef)* The exponential coefficients, also known as hazard ratios; *Se(coef)* The standard error of hazard ratios

In addition, time-dependent ROC analysis revealed that the performance of DFSPI was better than pathological TNM stage in predicting 3-year DFS of CRC patients (AUC 0.862 vs. 0.698) (Fig. 4b). Moreover, a prediction nomogram based on DFSPI and pathological TNM stage was developed to predict DFS for CRC patients (Fig. 4c). The C-index for this prediction nomogram was 0.841, and calibration curve demonstrated good consistency between prediction and observation of the nomogram (Fig. 4d and Additional file 1: Fig. S2). Furthermore, decision curve analysis demonstrated that the nomogram performed well in predicting the 3-year DFS rate in CRC patients (Fig. 4e).

### Validation of the ctDNA methylation marker-based prognostic prediction model

**Fig. 5:**
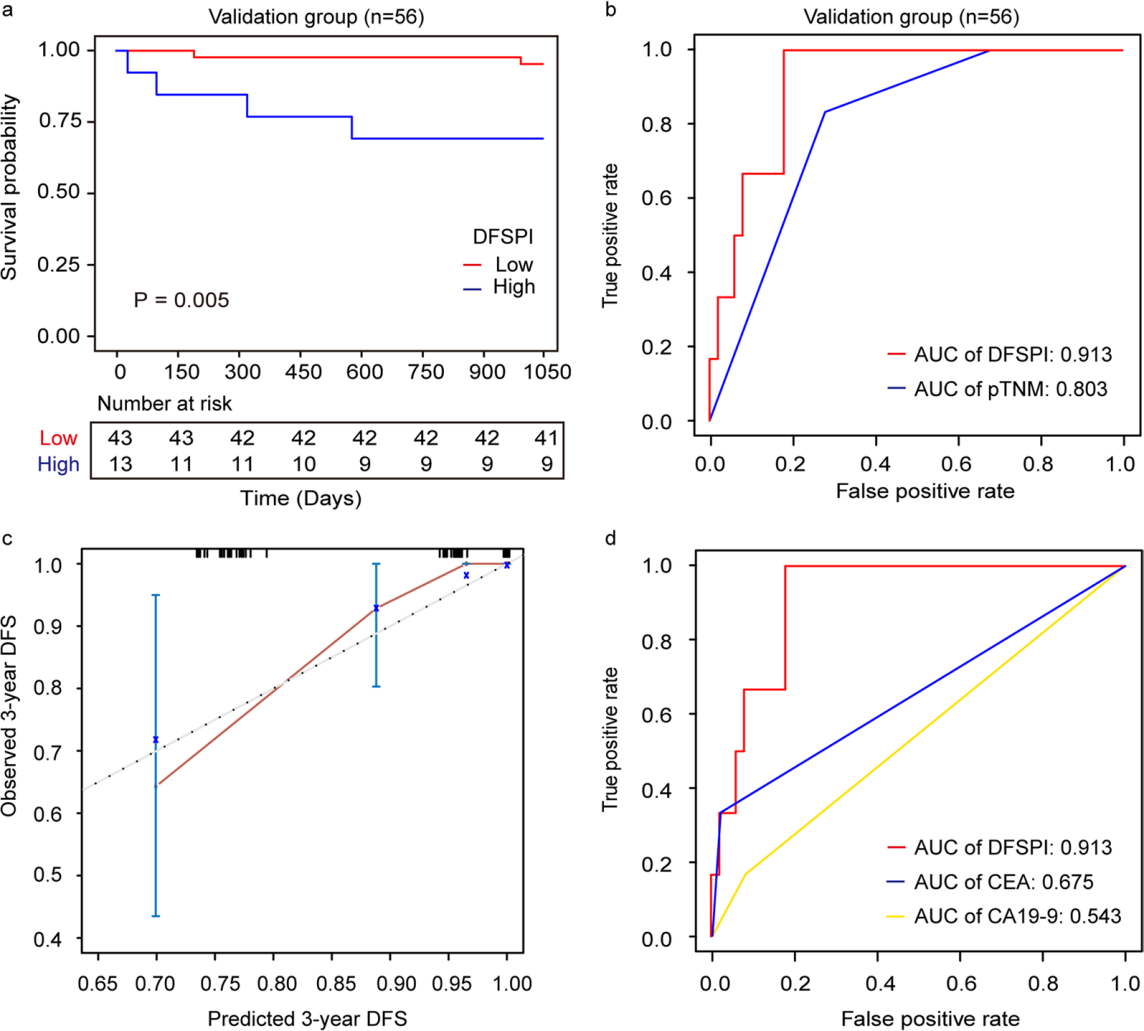
Validation of prognostic prediction model in CRC patients. **a** Survival curves of CRC patients separated by DFSPI in the validation group. **b** ROC and corresponding AUCs of DFSPI and pTNM in the validation group. **c** Calibration plot of nomogram for predicting 3-year DFS in the validation group. **d** ROC and corresponding AUCs for 3-year DFS predicted by DFSPI, CEA and CA19-9 in the validation group

To confirm the prognostic value of the ctDNA methylation marker-based prognostic prediction model, we applied the two-marker classifier in the validation cohort. Results showed that 43 (76.79%) patients were of low risk and 13 (23.21%) were of high risk. Univariate Cox regression analysis indicated that DFSPI was a risk factor for 3-year DFS, with HR as 7.796 [95% CI 1.425–42.660, *P* = 0.018]. Kaplan–Meier curves found that DFSPI could indicate patient prognosis (*P* = 0.005) (Fig. 5a). Time-dependent ROC analysis revealed that DFSPI was of superior performance than pathological TNM stage (AUC 0.913 vs. 0.803) (Fig. 5b). Furthermore, the calibration curve demonstrated that the prediction nomogram based on DFSPI and TNM stage was reliable and accurate for prognostic prediction (Fig. 5c). The ctDNA methylation model was also shown to be superior to CEA and CA19-9 in the validation cohort (AUC 0.913 versus 0.675 and 0.543, respectively) (Fig. 5d). Taken together, these results showed that ctDNA methylation marker could contribute to prognostic prediction in CRC patients.

### ctDNA-based overall survival prediction for CRC

To further evaluate the predictive performance of ctDNA-based diagnostic markers, we used Cox regression analysis to construct a prediction model and calculate a prognostic index for 3-year OS (OPI) using the 11 diagnostic markers (Additional file 1: Table S4). Unsupervised hierarchical clustering was used to show the association between 11 diagnostic markers and OS in the training and validation cohorts (Additional file 1: Fig. S3a, b). A combination of 11 diagnostic ctDNA markers could separate CRC patients into good prognosis versus poor prognosis group based on OS data from CRC patients in the training but not validation set (Additional file 1: Fig. S3c, d). A similar pattern was found in the ROC analysis (Additional file 1: Fig. S3e, f). These findings revealed the limited contribution of diagnostic ctDNA markers for OS prediction.

**Fig. 6:**
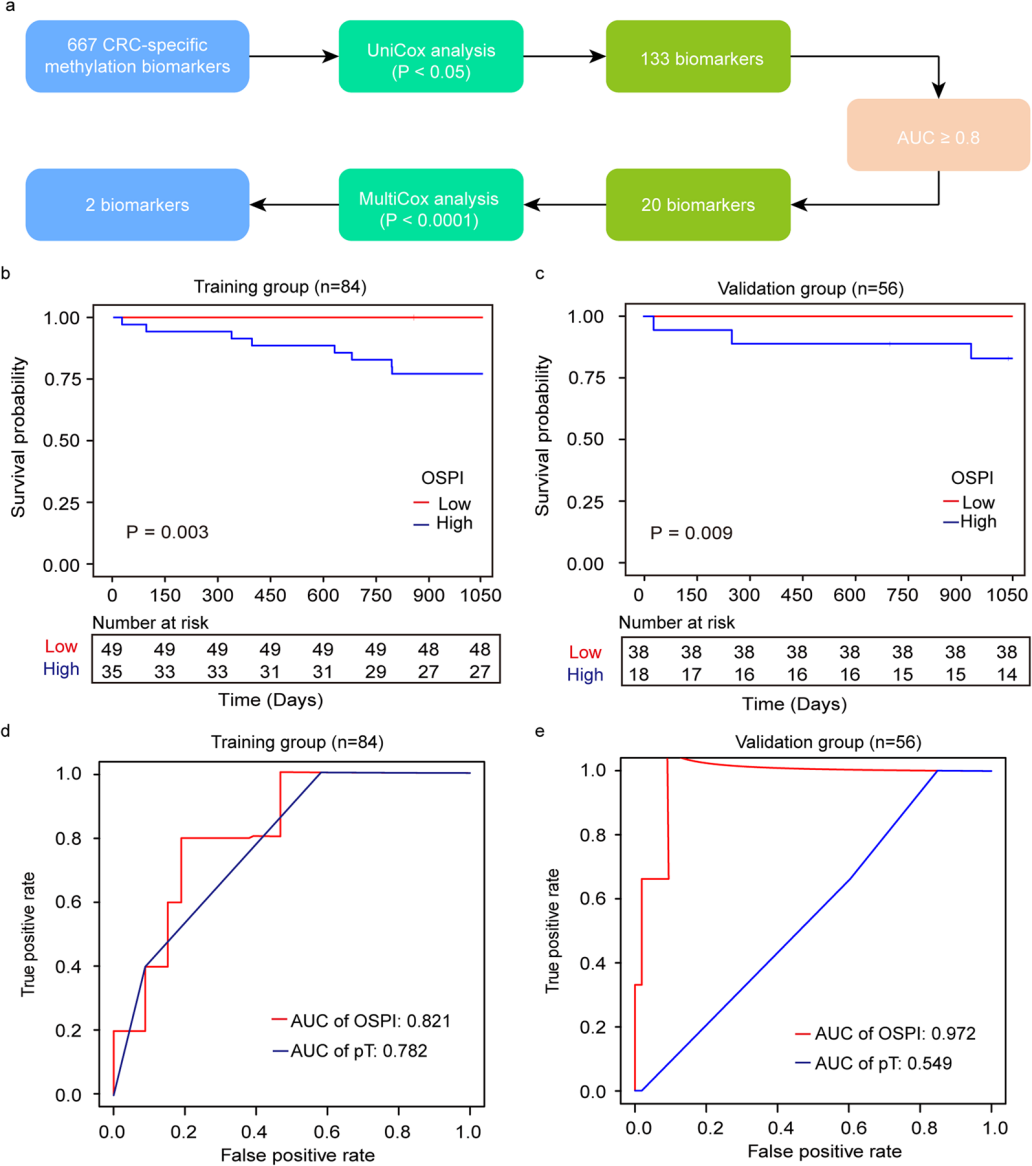
Prognostic prediction of 3-year OS based on ctDNA methylation markers. **a** Workflow for exploring 3-year OS related methylation markers. **b**–**c** 3-year OS curves of CRC patients with low OSPI and high OSPI in the training (**b**) and validation group (**c**). **d**–**e** ROC and corresponding AUCs for 3-year OS predicted by OSPI and pT in the training (**d**) and validation group (**e**)

In light of our above findings, we subsequently explored the potential of alternative methylation markers in predicting 3-year OS for CRC patients (Additional file 1: Table S5). Using the Cox regression model, the two-marker panel (cg05173737 and cg25224568) was used to construct a prognostic index for overall survival (OSPI) (Additional file 1: Table S6, Additional file 1: Fig. S4 and Fig. 6a). Univariate Cox regression analysis revealed that the OSPI was significantly associated with a higher risk of death within 3 years, with HR as 13.442 [95% CI 1.502–120.300, *P* = 0.020]. Kaplan–Meier curves showed that CRC patients with different OSPI had significantly different prognoses (training cohort: *P* = 0.003; validation cohort: *P* = 0.009) (Fig. 6b, c). Among the clinicopathological variables, the pathological T stage (*P* = 0.030) was significantly associated with OS in CRC patients (Table 3). Time-dependent ROC analysis showed that OSPI was better than pathological T stage in predicting the prognosis of CRC patients (training cohort: AUC, 0.821 vs. 0.782; validation cohort: AUC, 0.972 vs. 0.549) (Fig. 6d, e).

**Table 3:** Univariate Cox regression analysis with covariates including OSPI, and clinical characteristics for 3-year overall survival in the training group

Factor	coef	Exp(coef)	Se(coef)	z	*P*
OSPI	2.60	13.44	1.12	2.32	0.020
Age	0.08	1.09	0.05	1.61	0.108
Gender	− 1.77	0.17	1.12	− 1.58	0.114
BMI	− 0.22	0.80	0.91	− 0.25	0.806
CEA	− 17.06	3.90E−08	1.16E+04	− 0.001	0.999
CA19-9	0.52	1.68	1.12	0.46	0.643
pT	1.58	4.88	0.73	2.17	0.030
pN	0.23	1.26	0.64	0.37	0.714
pTNM	1.02	2.79	0.64	1.60	0.109

## Discussion

Despite current progress in cancer prognostic evaluation, the present prognostic assessment tool for CRC patients is still far from optimum due to limited efficacy. Computed tomography is routinely used for disease monitor, but it cannot detect minimal residual disease; the radiation characteristic further limits its frequent use as an instant test. These current situations highlight the need to identify new biomarkers that can effectively monitor the prognosis of CRC patients after radical surgery.

DNA methylation plays a vital role in tumorigenesis; it has been shown that ctDNA methylation status can be applied in the early diagnosis of multiple malignancies. The methylation of oncogenes and tumor suppressors presented at early stages of malignant transformation has been proved to exhibit significant values in cancer detection and diagnosis [31–36]. Our previous study [29] has established a promising non‐invasive diagnostic model based on the ctDNA methylation pattern (AUC: 0.91, sensitivity: 83.9%, specificity: 85.7%) for CRC patients. These findings suggest that DNA methylation patterns may play a pivotal role in malignancy recurrence prediction.

Considering the potential of ctDNA methylation in prognostic prediction, this work identified two markers for prognostic 3-year DFS prediction in CRC patients. According to their AUC values for 3-year DFS, it is conceivable that cg11186405 and cg17296166 might work together in CRC progression. The genomic locations of cg11186405 and cg17296166 indicate that they may affect the transcription of SOX1-OT and PNMA8A, respectively (Additional file 1: Table S7). Due to the lack of known functions of SOX1-OT and PNMA8A in CRC development, whether these two markers are connected with biological functions in CRC remains unknown. More importantly, it is of great clinical significance to explore the association between these markers and therapeutic resistance, which needs further investigation.

In this study, we constructed a prognostic prediction model (DFSPI) based on two ctDNA methylation markers (cg11186405 and cg17296166), which could stratify CRC patients with different prognoses. CEA and CA19-9 have been extensively utilized for routine surveillance in CRC patients. However, significant variations in CEA level can occur, and values exceeding 10 ng/mL can occasionally be observed without a clinically evident cause [37]. In our study, DFSPI demonstrated satisfactory performance in prognostic monitoring compared with CEA, CA19-9 and their combination (Fig. 3d–i). Consistent with previous studies [38, 39], the pathological TNM stage was identified as a predictor for 3-year DFS in this study. Moreover, the prognostic predictive power of DFSPI was superior to the pathological TNM stage. On the basis of these, a nomogram consisting of DFSPI and pathological TNM stage was constructed, which could contribute to identifying CRC patients who need more aggressive treatment and surveillance. The prediction accuracy of this nomogram was further supported by the satisfactory C-index and calibration.

Another prediction model (OSPI) was constructed using another two-marker panel (cg05173737 and cg25224568) to predict the 3-year OS in CRC patients. The OSPI was proved to be a prognostic risk factor to effectively distinguish CRC patients with different prognoses. The stratification potential of OSPI was superior to pathological T stage and conventional serum markers CEA and CA19-9 (Additional file 1: Fig. S4).

This study demonstrated that ctDNA methylation markers were associated with recurrence risk and survival in CRC patients after radical surgery. Based on these findings, a nomogram integrating pathological T stage and ctDNA methylation markers was developed to calculate the recurrence risk for each patient. This ctDNA methylation-based model is valuable for colorectal surgeons, which may help refine the postoperative recurrence risk calculation in CRC patients after radical surgery.

There are some inevitable limitations in this study. Firstly, the results of this study should be carefully interpreted due to the limited sample size. Secondly, the nomogram was established based on data from a single institution. Thirdly, a follow-up evaluation of these two markers is unavailable in the present work, and related studies are needed in future work. Further validation work from more medical centers would be ideal and necessary before the application in clinical practice.

## Conclusions

In summary, our findings show that the ctDNA methylation marker-based prognostic model can effectively stratify CRC patients into low and high recurrence risk groups. This model may add prognostic values to traditional clinicopathological risk factors in prognostic evaluation for CRC patients.

### Materials and methods

Detailed procedures are provided in Supplementary materials.

### Patients and samples

All specimens, including plasma, fresh–frozen tissues, and formalin-fixed paraffin-embedded (FFPE) tissues were collected at The Sixth Affiliated Hospital of Sun Yat-sen University (Guangzhou, China) from August 2016 to May 2018. The methylation data were assembled from 313 tissue samples [139 normal, 30 advanced adenomas (AA), and 144 CRC] and 577 plasma samples [169 healthy controls, 44 non-advanced adenomas (NAA), 76 AA, and 288 CRC], which were generated in our previous study [29]. All procedures were performed with the approval of the Institutional Review Board of the Sixth Affiliated Hospital of Sun Yat-sen University.

### Inclusion and exclusion criteria

The inclusion criteria were: (1) patients with CRC; (2) patients who underwent radical surgical resection in our center; and (3) patients' plasma undergoing DNA methylation sequencing. The exclusion criteria included: (1) patients with missing follow-up data; (2) patients with other cancers; (3) patients with distant metastases; (4) patients with follow-up < 3 years; and (5) patients with missing clinical data exceeding 20% of variables. Finally, 140 eligible CRC patients were included in this study and were randomly divided into a training group and a validation group at a 3:2 ratio.

### Definition and variables

Clinical variables and demographic were defined as follows: gender, age at the time of surgery, body mass index (BMI), elevated CEA level (> 100 ug/L), elevated CA19-9 level (> 37 U/mL), and pathological TNM staging.

### Statistical analysis

Frequencies were used to describe continuous factors, and means and standard deviations were applied to categorical factors. The chi-square test for categorical variables and a two-tailed *t* test for continuous variables were used to test baseline balance between the training and validation groups. The Cox regression model was used to analyze the correlation between ctDNA methylation markers and DFS or OS. Time-dependent ROC analysis was used to shrink the number of biomarkers. Univariate Cox analysis was applied to screen prognostic-related clinical indicators. The Cox regression model was utilized in the univariate survival analysis, and Cox regression coefficients were used for nomogram construction. Decision curve analysis was conducted to determine the effectiveness of the nomogram by quantifying the net benefits at different threshold probabilities. The *P* value less than 0.05 was considered statistically significant. The *R* software (version 4.2.1; http://www.r-project.org) was used for all analysis.

## Supplementary Information

**Additional file 1.  Supplemental Figures and Tables.**

## Data Availability

The datasets used and analyzed during the current study are available from the corresponding author upon reasonable request.

## Abbreviations

CRC: Colorectal cancer
DFS: Disease-free survival
HR: Hazard ratio
CI: Confidence interval
CEA: Carcinoembryonic antigen
CA19-9: Carbohydrate antigen19-9
ctDNA: Circulating tumor DNA
FFPE: Formalin-fixed paraffin-embedded
AA: Advanced adenoma
NAA: Non-advanced adenoma
BMI: Body mass index
OS: Overall survival
ROC: Receiver operating characteristic
DPI: Prognostic index for DFS by diagnostic markers
AUC: Area under receiver operating characteristic curve
DFSPI: Prognostic index of the 3-year DFS
OPI: Prognostic index for OS by diagnostic markers
OSPI: Prognostic index of overall survival
