# Supplementary material for: Predicting disease-free survival in colorectal cancer by circulating tumor DNA methylation markers
Source: Clin Epigenetics. 2022 Dec 1;14:160. doi: 10.1186/s13148-022-01383-8 (PMC9714195; doi:10.1186/s13148-022-01383-8)
Supplement: Supplementary file 1 — Additional file 1. Supplemental Figures and Tables. [file 13148_2022_1383_MOESM1_ESM.pdf]

Supplementary Materials for

Predicting Disease-free Survival in Colorectal Cancer by Circulating Tumor DNA Methylation Markers

Xin Yang, Xiaofeng Wen, Qin Guo, Yunfeng Zhang, Zhenxing Liang, Qian Wu, Zhihao Li, Weimei Ruan, Zhujia Ye, Hong Wang, Zhiwei Chen, Jian-Bing Fan, Ping Lan,Huashan Liu, Xianrui Wu

Correspondence to: wuxianr5@mail.sysu.edu.cn

This PDF file includes:

Supplementary Figure 1 to 4

Supplementary Table 1 to 7

Supplementary Materials and Methods

Supplementary Figures

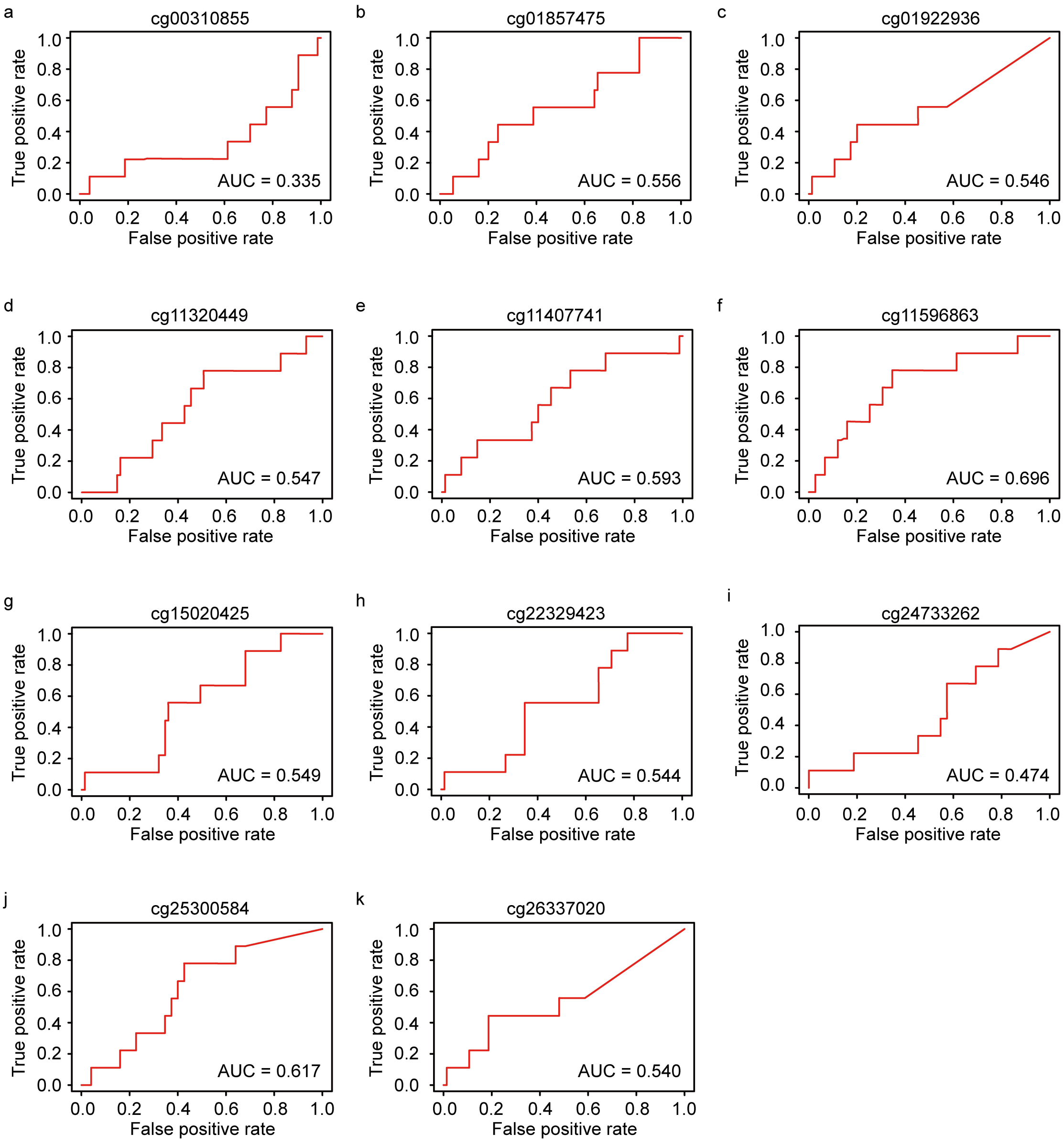

Fig. S1 Receiver operating characteristic curves of the 11 diagnostic markers (a to k) Receiver operating characteristic (ROC) curves and the associated areas under curves (AUCs) of the 11 diagnostic markers using ctDNA methylation analysis in the training group.

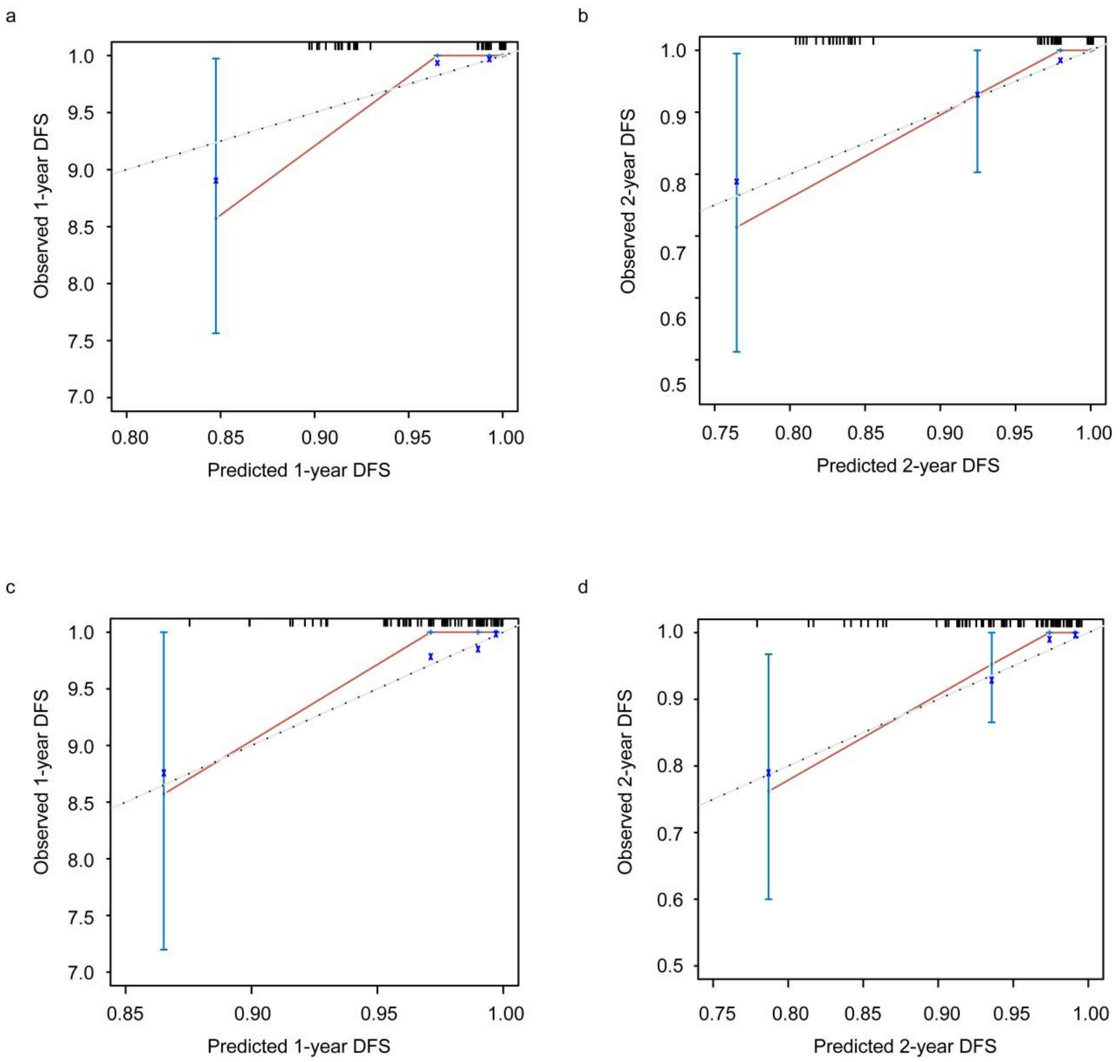

Fig. S2 Calibration plot of nomogram for DFS based on ctDNA methylation markers. (a-b) Calibration plot of nomogram for predicting one- and two-year disease-free survival in the training group. (c-d) Calibration plot of nomogram for predicting one- and two-year disease-free survival in the validation group.

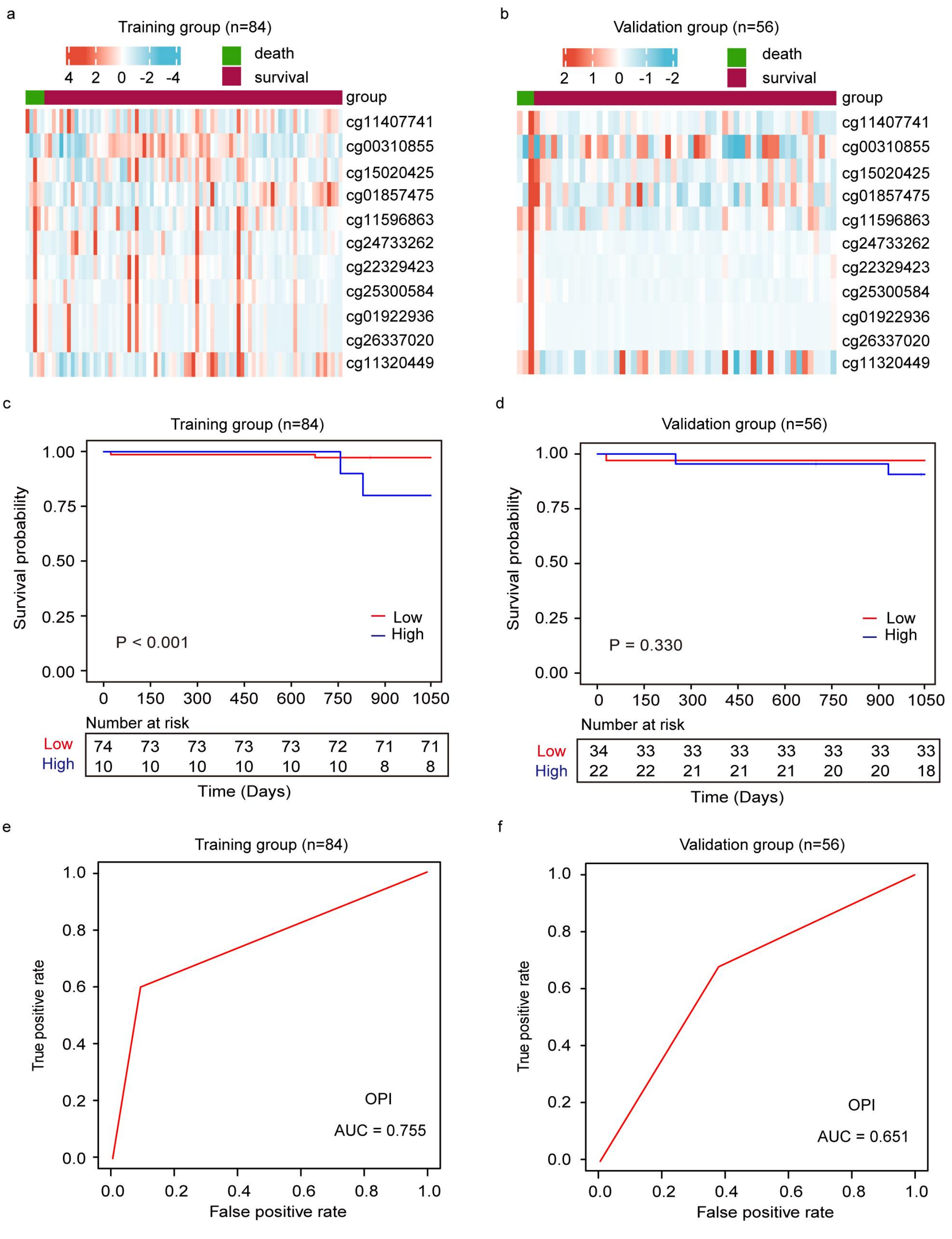

Fig. S3 Prognostic prediction of 3-year OS based on diagnostic markers. Unsupervised hierarchical clustering of the diagnostic markers in the training (a) and validation group (b). 3-year OS curves of patients with CRC with low or high risk according to the prognostic index of diagnostic markers (OPI) in the training group (c) and validation group (e). ROC and corresponding AUCs for 3-year OS predicted by OPI in the training group (d) and validation group (f).

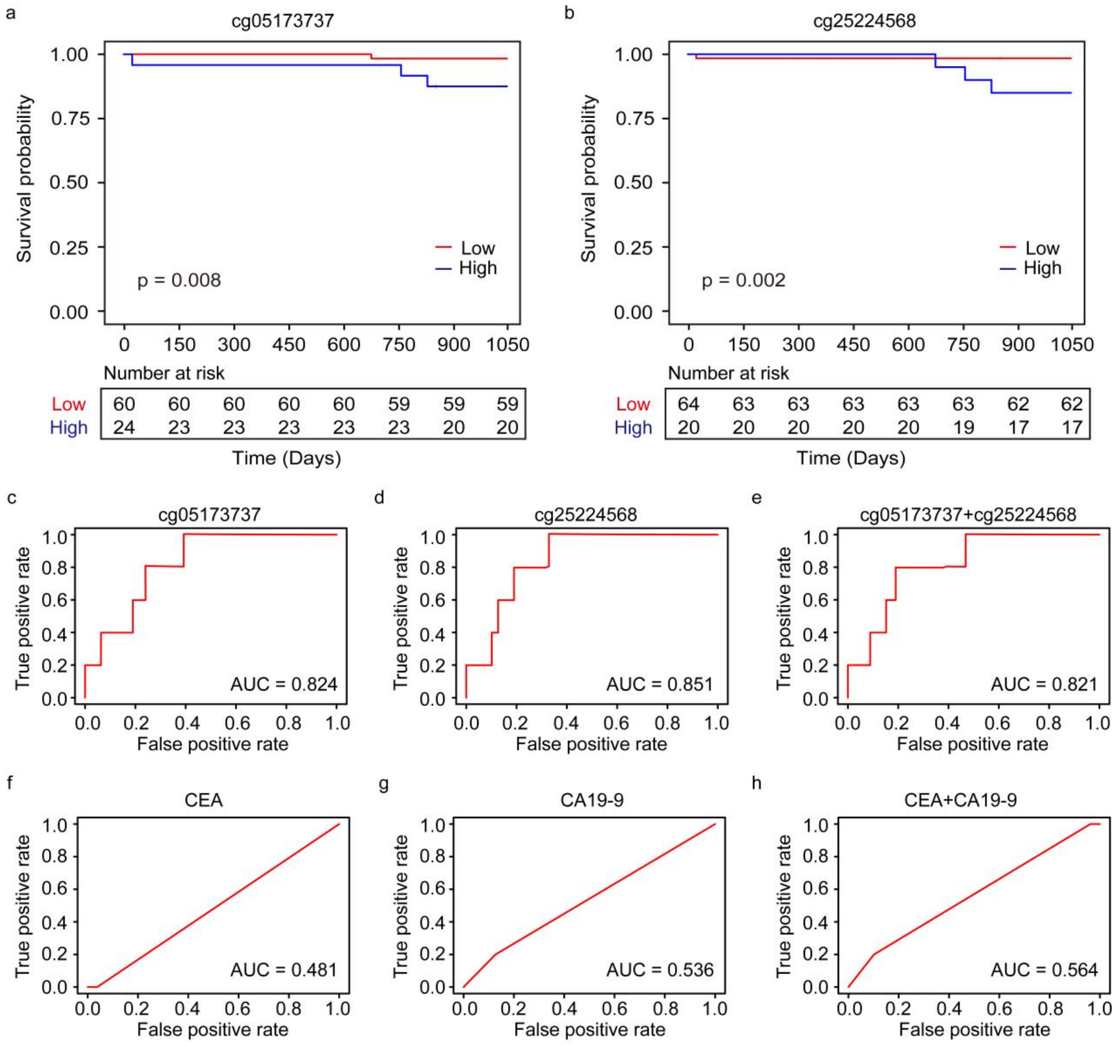

Fig. S4 Survival analysis for the biomarkers related to 3-year OS. (a-b) 3-year OS curves of patients with CRC with low or high risk according to cg05173737 (a) and cg25224568 (b) in the initial group. (c-e) ROC and corresponding AUCs for 3-year OS predicted by cf-DNA methylation biomarkers of the 3-year OS prediction model in the training group. (f-h) ROC and corresponding AUCs for 3-year OS predicted by CEA, CA19-9 and both combined in the training group.

Supplementary Tables

Table S1 Characteristics of the diagnostic markers and their coefficients for 3-year disease-free survival in the training group.

**Methylation marker**	**coef**	**Exp(coef)**	**Se(coef)**	**z**	***P*-value**
**cg11407741**	2.18	8.80	16.65	0.13	0.896
**cg00310855**	-12.16	5.24E-06	6.63	-1.83	0.067
**cg15020425**	1.44	4.23	13.65	0.11	0.916
**cg01857475**	-4.67	9.40E-03	8.24	-0.57	0.571
**cg11596863**	25.88	1.73E+11	11.98	2.16	**0.031**
**cg24733262**	-49.23	4.17E-22	47.67	-1.03	0.302
**cg22329423**	82.33	5.71E+35	64.43	1.28	0.201
**cg25300584**	-44.09	7.14E-20	30.25	-1.46	0.145
**cg01922936**	579.40	4.08E+250	727.50	0.80	0.426
**cg26337020**	-594.80	4.593E-259	731.50	-0.81	0.416
**cg11320449**	-6.32	1.81E-03	9.61	-0.66	0.511

DPI=(2.18*cg11407741)-(12.16*cg00310855)+(1.44*cg15020425)-(4.67*cg01857475)+(25.88*cg11596863)-(49.23*cg24733262)+(82.33*cg22329423)-(44.09*cg25300584)+(579.4*cg01922936)-(594.8*cg26337020)-(6.32*cg11320449).

Table S2 Characteristics of 55 methylation markers with *P* < 0.05 using univariate Cox regression analysis among 667 CRC-specific DNA methylation biomarkers.

**Methylation marker**	**Uni-cox analysis** *P*-value	**AUC**	**Multi-cox analysis** *P*-value
cg13574390	**0.026**	**0.729**	0.145
cg08839858	**0.050**	0.582	
cg15193782	**0.008**	**0.735**	0.538
cg20209956	**0.001**	0.686	
cg19021466	**0.015**	**0.820**	0.189
cg11263393	**0.002**	0.511	
cg26798879	**0.045**	0.555	
cg11186405	**0.007**	**0.742**	**0.049**
cg08252855	**0.017**	0.623	
cg04234680	**0.002**	0.610	
cg15897970	**0.014**	0.674	
cg05173737	**0.026**	**0.700**	0.360
cg11797092	**0.039**	0.551	
cg01667646	**0.025**	0.629	
cg09359907	**0.011**	0.691	
cg01653005	**0.011**	0.672	
cg21139392	**0.006**	0.511	
cg11416076	**0.021**	0.550	
cg21521784	**0.026**	**0.723**	0.435
cg24588375	**0.042**	0.617	
cg02711801	**0.047**	0.617	
cg17296166	**0.001**	**0.829**	**0.021**
cg23547515	**0.019**	0.562	
cg11740878	**0.009**	0.588	

cg14277392	**0.031**	0.599	
cg17849956	**0.009**	0.664	
cg00397851	**0.008**	0.667	
cg20607331	**0.040**	0.615	
cg16697731	**0.044**	0.611	
cg17684296	**0.005**	0.639	
cg13552710	**0.028**	0.480	
cg25999722	**0.024**	0.595	
cg14163665	**0.029**	0.659	
cg22762844	**0.017**	0.623	
cg11912765	**0.040**	**0.752**	0.571
cg24463006	**0.029**	0.571	
cg00651829	**0.020**	0.617	
cg13348059	**0.019**	0.638	
cg03175305	**0.007**	**0.761**	0.325
cg13542964	**0.016**	0.661	
cg20772101	**0.036**	0.475	
cg13562911	**0.033**	0.642	
cg10883284	**0.010**	0.513	
cg14774364	**0.027**	**0.750**	0.090
cg25966893	**0.038**	**0.729**	0.565
cg06554200	**0.024**	0.683	
cg13070193	**0.011**	**0.725**	0.828
cg06248179	**0.006**	**0.856**	0.091
cg07596524	**0.038**	0.696	
cg20079899	**0.005**	**0.714**	0.602
cg20675505	**0.028**	0.646	
cg25224568	**0.004**	0.659	
cg21639387	**0.010**	0.534	

cg13462843	**0.010**	0.554	
cg05124235	**0.008**	**0.768**	0.057

Table S3 Characteristics of the two methylation markers which conducted DFSPI and their coefficients for 3-year disease-free survival in the training group.

**Methylation marker**	**coef**	**HR**	**CI(lower)**	**CI(upper)**	**z**	***P*-value**
**cg11186405**	14.35	1.71E+06	2.89E-14	1.01E+26	0.62	0.537
**cg17296166**	19.96	4.67E+08	5.17E-02	4.21E+18	1.71	0.088

HR: The hazard ratios; **CI**: The confidence interval.

Table S4 Characteristics of the diagnostic markers and their coefficients for 3-year overall survival in the training group.

Methylation marker	coef	Exp(coef)	Se(coef)	z	*P*-value
**cg11407741**	-0.92	0.40	22.68	-0.04	0.968
**cg00310855**	-25.18	1.16E-11	12.94	-1.95	0.052
**cg15020425**	7.38	1606.00	20.66	0.36	0.721
**cg01857475**	-0.41	0.66	9.72	-0.04	0.966
**cg11596863**	21.63	2.48E+09	18.76	1.15	0.249
**cg24733262**	-20.95	7.94E-10	67.61	-0.31	0.757
**cg22329423**	91.16	3.90E+39	112.50	0.81	0.418
**cg25300584**	-37.50	5.17E-17	43.66	-0.86	0.390
**cg01922936**	160.30	4.13E+69	903.60	0.18	0.859
**cg26337020**	-209.90	7.26E-92	901.50	-0.23	0.816
**cg11320449**	7.49	1.78E+03	13.10	0.57	0.568

Table S5 Characteristics of 20 methylation markers (*P* < 0.05 and AUC ≥ 0.8) among 667 CRC-specific DNA methylation biomarkers.

Methylation marker	Uni-cox analysis *P* < 0.05	AUC ≥ 0.8	Multi-cox analysis *P*-value
cg05140069	**0.022**	**0.826**	0.0238
cg15193782	**0.001**	**0.884**	0.0003
cg19021466	**0.006**	**0.895**	0.7080
cg11186405	**0.002**	**0.839**	0.1412
cg03502284	**0.043**	**0.808**	0.3581
cg05173737	**0.008**	**0.824**	**<0.0001**
cg09359907	**0.002**	**0.844**	0.0018
cg13380624	**0.026**	**0.836**	0.8855
cg21521784	**0.004**	**0.883**	0.9126
cg17296166	**0.005**	**0.801**	0.0685
cg22795590	**0.018**	**0.839**	0.8975
cg00651829	**0.001**	**0.841**	0.0680
cg03175305	**0.001**	**0.905**	0.1305
cg06848047	**0.036**	**0.876**	0.0421
cg25966893	**0.007**	**0.874**	0.8086
cg12926104	**0.049**	**0.867**	0.6228
cg06248179	**0.006**	**0.876**	0.0043
cg20079899	**0.003**	**0.816**	0.0170
cg25224568	**0.002**	**0.851**	**<0.0001**
cg05124235	**0.007**	**0.841**	0.0018

Table S6 Characteristics of the two methylation markers which conducted OSPI and their coefficients for 3-year overall survival in the training group.

Methylation marker	coef	HR	CI(lower)	CI(upper)	z	*P*-value
**cg05173737**	-5.34	4.82E-03	8.28E-61	2.80E+55	-0.08	0.937
**cg25224568**	20.99	1.31E+09	2.95E-13	5.82E+30	0.83	0.409

Table S7 Characteristics of the 2 methylation markers in ctDNA methylation.

Methylation marker	Chromosome	Start	End	Gene
**cg11186405**	Chromosome 13	112728105	112728306	SOX1-OT
**cg17296166**	Chromosome 19	46974466	46974667	PNMA8A

Supplementary Materials and Methods

Tissue samples

The tumor tissues and the corresponding adjacent normal tissues were collected from patients receiving colorectal cancer (CRC) resection. The normal intestine which were more than 5 cm away from the primary tumor were selected as the adjacent normal tissues. The tissue samples of advanced adenoma (AA) were obtained from formalin-fixed paraffin-embedded (FFPE) specimens from The Sixth Affiliated Hospital of Sun Yat-sen University.

Plasma samples

10 ml blood was drawn from healthy controls or treatment-naive patients using BD Vacutainer® EDTA Tubes (Becton, Dickinson and Company, Cat# 367525) and plasma was immediately separated within 2 h after blood draw and stored at -80 ℃ for a median of 9 days (range: 1 to 35 days) until DNA isolation and subsequent assays. The resultant plasma volume ranged from 2.0 ml to 3.2 ml. The healthy controls consisted of patients with benign anorectal diseases, such as hemorrhoids, anal fissure, and perianal fistula.

Isolation of tissue genomic DNA and plasma cell-free DNA

Tissue genomic DNA was isolated from fresh frozen and FFPE tissue samples using the Qiagen DNeasy Blood & Tissue Kit (Qiagen, Cat#: 69504) and the QIAamp DNA FFPE Tissue Kit (Qiagen, Cat# 56404), respectively. Cell-free DNA (cfDNA) was isolated from plasma using the Bioo NextPrep-Mag™ cfDNA Isolation Kit (Bioo Scientific, Austin, TX, USA, Cat# NOVA-3825). Repeated freezing and thawing of plasma were avoided to prevent cfDNA degradation. The concentration and quality of cfDNA were determined using the Qubit™ dsDNA HS Assay Kit (Thermo Fisher Scientific, Eugene, OR, USA, Cat# Q32854) and the Agilent High Sensitivity DNA Kit (Agilent, Waldbrann, Germany, Cat# 5067-4626) on a 2100 Bioanalyzer Instrument (Agilent), which assessed the size distribution of cfDNA. The cfDNA with yield greater than 3 ng and without overt genomic DNA contamination was used for

sequencing library construction.

Bisulfite conversion

Bisulfite conversion was performed using the Zymo Lightning Conversion Reagent (Zymo Research, Irvine, CA, USA, Cat# D5031) according to the manufacturer's protocol. For tissue samples, 2 μg of genomic DNA was fragmented into ~200 bp fragments (peak size) by a M220 Focused-ultrasonicator (Covaris, Inc, Boston, MA, USA) following the manufacturer's instructions, and 800 ng of purified fragmented genomic DNA was then used for the following bisulfite conversion. After bisulfite conversion, the purified bisulfite-converted DNA was quantified at A260 by NanoDrop (Thermo Fisher Scientific). Then, 100 and 150 ng of the bisulfite-converted products were applied for library preparation for fresh frozen and FFPE tissue samples, respectively. For plasma samples, the recommended input of cfDNA for bisulfite conversion is 10 ng. If cfDNA yield was between 3 and 10 ng, all the purified cfDNA was used for bisulfite conversion. After bisulfite conversion, we used all the bisulfite-converted cfDNA for library preparation without DNA quantification to avoid cfDNA loss. Following DNA bisulfite conversion, the bisulfite-converted DNA was run through a Zymo-Spin™ IC Column, washed and desulfonated, and then eluted twice using the M-Elution buffer to a final volume of 17 μl.

AnchorIRIS™ pre-library construction

AnchorIRIS™ (Guangzhou, Guangdong) pre-hyb library construction was performed using AnchorDx EpiVisio™ Methylation Library Prep Kit (AnchorDx, Guangzhou, China, Cat# A0UX00019) and AnchorDx EpiVisio™ Indexing PCR Kit (AnchorDx, Cat# A2DX00025). Following the procedure of end pair reparation, 3' end adaptor ligation, and amplification of reverse complement DNA (Liang *et al.* [1]), the amplified DNA was purified using 1:6 Agencourt AMPure XP Magnetic Beads (Beckman Coulter, Brea, CA, USA, Cat# A63882). After 3' end adaptor ligation of reverse complement DNAs and indexing PCR (i5 and i7; Liang *et al.* [1]), the

amplified pre-libraries were subsequently purified using XP Magnetic Beads. Pre-hyb libraries containing more than 800 ng DNA were used for target enrichment assay.

AnchorIRIS^{TM} target enrichment

Target Enrichment was performed using AnchorDx EpiVisio^{TM} Target Enrichment Kit (AnchorDx, Cat# A0UX00031) and methylation panels, AnchorDx PanMet V1 or V2. A total of 1,000 ng of DNA containing up to 4 pre-hyb libraries was pooled for target enrichment using AnchorDx PanMet V1 or V2 methylation panels. AnchorDx PanMet V2 included 12624 pre-selected regions enriched for cancer-specific methylation that contained all the 9921 regions of AnchorDx PanMet V1. The total sizes of the genomic regions targeted by the AnchorDx PanMet V1 and V2 panels are 563,272 and 733,057 bp, which cover 45,566 and 55,369 CpG sites, respectively. Probe hybridization, purification, and final PCR amplification were carried out according to the protocol from Liang [1].

DNA methylation level calculation

Enriched libraries were sequenced by Illumina HiSeq X Ten Sequencing System. Percentage of co-methylated reads (PCM) was calculated by the analysis pipeline developed by Liang [1].

$$\mathrm{PCM} = \frac{\#\ \mathrm{co} - \mathrm{methylated\ reads\ of\ a\ region}}{\#\ \mathrm{all\ mapped\ reads\ with\ at\ least\ 3\ CpGs\ in\ the\ region}}$$

Reads having at least 3 methylated CpGs within a sliding window of 5 CpGs were designated as co-methylated reads that were used for subsequent analysis of methylation patterns and predictive modeling of malignant/normal states of patient samples. Log2 PCM was used for the model construction to optimize the model's performance and stability.

Obtained CRC-specific cfDNA methylation biomarkers

With the AnchorDx PanMet methylation panels, we first performed a differential methylation analysis on normal, advanced adenoma and CRC tissue samples using

Wilcoxon signed-rank test, then the *P*-value for each methylation biomarker was then corrected by multiple testing by the Benjamini-Hochberg Procedure (BHP) to control false discovery rate at a significance level of 0.05. We also calculated the distinguishing power by the area under receiver operating characteristic curve (AUROC) and absolute methylation change for each biomarker. CRC-specific methylation biomarkers were identified using the following criteria: 1) significant difference between CRC and normal tissue samples (adjusted $P < 0.05$) with relatively large absolute change (> 0.2); 2) significant difference between advanced adenoma and normal tissue samples (adjusted $P < 0.05$) with relatively large absolute change (> 0.2); 3) same trend for CRC and advanced adenoma compared to normal controls. We also added methylation biomarkers which were significantly different between advanced adenoma and CRC to improve the potential differentiation power. In total, 667 CRC-specific DNA methylation biomarkers were obtained in this tissue cohort analysis, and apply them to do the further analysis in the plasma cohort.

References

1. Liang W, Zhao Y, Huang W, Gao Y, Xu W, Tao J, et al. Non-invasive diagnosis of early-stage lung cancer using high-throughput targeted DNA methylation sequencing of circulating tumor DNA (ctDNA). Theranostics. 2019;9(7):2056-2070.
